# Unfolded Protein Response Signaling in Liver Disorders: A 2023 Updated Review

**DOI:** 10.3390/ijms241814066

**Published:** 2023-09-14

**Authors:** Smriti Shreya, Christophe F. Grosset, Buddhi Prakash Jain

**Affiliations:** 1 Gene Expression and Signaling Lab, Department of Zoology, Mahatma Gandhi Central University, Motihari 845401, Bihar, India; smritishreyasss@gmail.com; 2 MIRCADE Team, U1312, Bordeaux Institute in Oncology, BRIC, Université de Bordeaux, 146 Rue Léo Saignat, F-33000 Bordeaux, France

**Keywords:** endoplasmic reticulum, unfolded protein response, liver disorders, non-alcoholic fatty liver disease, hepatocellular carcinoma

## Abstract

Endoplasmic reticulum (ER) is the site for synthesis and folding of secreted and transmembrane proteins. Disturbance in the functioning of ER leads to the accumulation of unfolded and misfolded proteins, which finally activate the unfolded protein response (UPR) signaling. The three branches of UPR—IRE1 (Inositol requiring enzyme 1), PERK (Protein kinase RNA-activated (PKR)-like ER kinase), and ATF6 (Activating transcription factor 6)—modulate the gene expression pattern through increased expression of chaperones and restore ER homeostasis by enhancing ER protein folding capacity. The liver is a central organ which performs a variety of functions which help in maintaining the overall well-being of our body. The liver plays many roles in cellular physiology, blood homeostasis, and detoxification, and is the main site at which protein synthesis occurs. Disturbance in ER homeostasis is triggered by calcium level imbalance, change in redox status, viral infection, and so on. ER dysfunction and subsequent UPR signaling participate in various hepatic disorders like metabolic (dysfunction) associated fatty liver disease, liver cancer, viral hepatitis, and cholestasis. The exact role of ER stress and UPR signaling in various liver diseases is not fully understood and needs further investigation. Targeting UPR signaling with drugs is the subject of intensive research for therapeutic use in liver diseases. The present review summarizes the role of UPR signaling in liver disorders and describes why UPR regulators are promising therapeutic targets.

## 1. Introduction

The endoplasmic reticulum (ER) is one of the largest organelles found in all eukaryotic organisms. It is responsible for the synthesis and folding of secreted and transmembrane proteins and serves as an important site for intercellular calcium reservoirs and the post-translational modification of secreted and membrane proteins [1]. It is also an important site for lipid synthesis, hormone production, and drug metabolism.

Proteins residing in the ER, together with the proteins targeted for Golgi and plasma membrane, are synthesized by ribosomes attached to the ER membrane. The newly synthesized polypeptide contains a signal sequence at the N-terminal, which is further recognized by signal recognition particles (SRPs). This signal recognition protein along with the nascent polypeptide and ribosome binds to the α-subunit of the SRP receptor in the ER membrane. This SRP has two functions: it directs the binding of the ribosome to the ER membrane and also the insertion of newly synthesized proteins into the transmembrane channel. This newly synthesized protein chain continues to elongate inside the ER lumen with the help of a translocation channel called the translocon. The movement of growing polypeptides through the translocon is an ATP-driven process. Once the protein enters the ER lumen, the signal sequence is cleaved with the help of signal peptidase and the translocated polypeptide then undergoes post-translational modifications and folding to gain its appropriate three-dimensional shape and function [2].

Several important modifications occur inside the ER lumen, including the formation of the disulfide bond, an important covalent connector for the tertiary and quaternary shape of the proteins. Proper folding of proteins, addition, and processing of carbohydrates, accurate proteolytic cleavage, and the correct assembly of multimeric proteins are important steps occurring inside the ER lumen. Its unique oxidizing environment plays a key role in protein folding. Protein disulphide-isomerase (PDI) helps in the formation of the disulfide bond and its proper formation and localization. The addition and processing of carbohydrates, i.e., glycosylation, is a major chemical modification for most of the proteins synthesized. These carbohydrate chains in glycoproteins become attached to either the hydroxyl group in serine and threonine residues forming O-linked oligosaccharides or to the amide nitrogen of asparagine forming N-linked oligosaccharides [2,3].

Chaperones are a class of proteins that bind to the unfolded proteins and improve the protein folding efficiency of the newly synthesized polypeptide chains. The most abundant proteins inside the ER lumen are HSP70 family member chaperones including BiP, which is encoded by the *HSPA5* gene and is also known as 78 kDa glucose-regulating protein 78 (GRP78). Calnexin and Calreticulin, which are calcium-binding proteins, facilitate the proper folding of multimeric proteins [3]. Calcium plays an important role in protein folding as it acts as a cofactor for various chaperones. Hence, the ER acts as quality control of proteins, thereby guaranteeing that only correctly folded proteins exit it [4].

The ER also acts as a calcium reservoir. Hence, ER homeostasis is crucial for cell function and survival. Any disturbance in the functioning of ER homeostasis compromises the protein folding capacity of ER, leading to an accumulation of unfolded or misfolded proteins inside the ER lumen. However, the ER tries to maintain this protein folding capacity. If unable to do so, it undergoes stress. If a polypeptide chain fails to gain its native structure, it undergoes degradation through a pathway called ERAD (ER-associated degradation), which is the retro-translocation of misfolded proteins into the cytosol from the ER and further degradation by 26S proteasome. If the proteins somehow regain their original shape, they are further translocated to the Golgi and continue the secretory pathway [5].

ER plays a crucial role in membrane and lipid synthesis and fat accumulation for energy storage. It contains several host enzymes which catalyze the synthesis of membrane lipids. Since ER stores members of the sterol regulatory element-binding protein family, it monitors cholesterol homeostasis [4].

In response to ER stress, cells activate certain adaptive mechanisms that help to overcome it and maintain ER homeostasis. Various factors can disturb ER functioning including a cell-autonomous mechanism consecutive to oncogene activation, which alters the homeostatic equilibrium of the ER. Hypoxia, starvation, nutrient deprivation, glucose starvation, temperature variation, ROS, and free radicals all induce ER stress [6].

ER stress can also be induced by ER inducers such as tunicamycin, which blocks the biosynthesis of glycoprotein in the ER, causing the accumulation of unfolded or misfolded proteins. Dithiothreitol disturbs ER functioning by blocking protein disulfide bond formation. Another ER stress inducer is brefeldin A, which disturbs protein trafficking from ER to Golgi and enhances the retrograde transport of proteins from Golgi to ER, leading to the accumulation of misfolded proteins inside the ER lumen. In addition, thapsigargin reduces the calcium concentration inside the ER, which inhibits the activity of calcium-dependent ER chaperones by inhibiting the sacro/endoplasmic reticulum (ER) Ca^2+^ ATPase (SERCA) and hence reduces the protein-folding capacity [7,8].

ER stress and UPR signaling have received considerable attention in various neurodegenerative and non-neurodegenerative diseases including diabetes, vascular stroke, cancer, and heart disease. Many neurodegenerative diseases like Parkinson’s disease and Alzheimer’s disease are characterized by the deposition of misfolded proteins in neurons [9]. Calcium ions are known to play an important role in neuronal functions. ER stress can disturb calcium homeostasis [10]. It can also activate several inflammatory pathways and contribute to the pathogenesis of diseases. It can promote cancer progression and angiogenesis in the tumor microenvironment [11].

## 2. UPR Signaling Cascade

**Figure 1:**
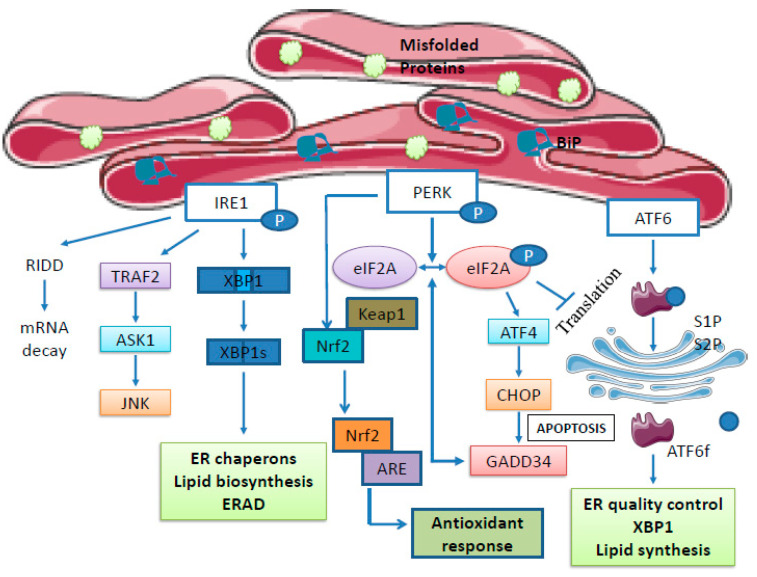
The three branches of Unfolded Protein Response Signaling.

To restore the protein-folding capacity, the unfolded protein response is activated and mainly coordinated by precise transcriptional and translational processes within the cell. The UPR monitors the protein folding level and maintains ER homeostasis [12]. This accumulation of unfolded or misfolded proteins is sensed by three major biosensors (transmembrane receptors) referred to as inositol-requiring enzyme 1 (IRE1), protein kinase RNA-activated (PKR)-like ER kinase (PERK), and activating transcription factor 6 (ATF6) [13]. These sensors are structured in such a manner that they harbor three major domains, including a cytosolic domain, a single-pass membrane-spanning domain, and an ER luminal domain. In normal conditions, these sensors are bound to ER resident chaperons, BiP or GRP78, which maintains them in an inactive state. As misfolded/unfolded proteins accumulate, BiP and GRP78 chaperones become detached from their sensors, bind the accumulated unfolded proteins, and finally activate the sensors [14]. Various UPR pathways are represented in Figure 1.

### 2.1. PERK Signaling

PERK is a trans-membrane protein embedded inside the ER membrane. In normal conditions, the biosensor PERK is bound to chaperone GRP78, which prevents the dimerization of PERK with other PERK proteins. The accumulation of unfolded proteins results in the release of GRP78 from PERK and its attachment to unfolded proteins. The ER luminal domain of PERK then undergoes oligomerization and trans-autophosphorylation, thus activating PERK. This activated PERK further phosphorylates eukaryotic initiation factor 2 alpha (eIF2α) at serine 51. Because this initiation factor regulates the initial step of translation by binding tRNA to the 40S ribosomal subunit, phosphorylation of eIF2α inhibits general translation and, consequently, reduces the loading of ER by newly synthesized proteins [15]. It decreases the overall protein synthesis and thereby helps cells to respond to various stress conditions. When eIF2α is limited, it selectively binds to the short open reading frame present in the 5′UTR of the *ATF4* (activating transcription factor 4) gene and selectively increases its expression. ATF4 is a transcription factor that migrates to the nucleus and increases the expression of various chaperones and UPR target genes [16,17]. It also transcriptionally activates the expression of the *DDIT3* gene, which translates into CHOP protein. CHOP activates the apoptotic pathway by down-regulating the expression of the B-cell lymphoma 2 (BCL2) protein which is anti-apoptotic [18]. CHOP is also a transcriptional activator of growth arrest and DNA damage-inducible protein (GADD34). GADD34 acts in a negative feedback loop and dephosphorylates eIF2α to restore the translation process [19]. Normally, nuclear erythroid factor 2 (NRF2) is present in its inactive form in the cytoplasm through its association with KEAP1. If ER stress occurs, PERK phosphorylates NRF2 causing its dissociation from KEAP1, its nuclear localization, and the activation of gene transcription by binding the antioxidant response element (ARE). This ARE controls the expression of genes that are involved in oxidative response like glutathione S-transferase and UDP-glucosyl transferase, as well as the metabolism of xenobiotics, thus preserving cells against oxidative stress during ER stress [20,21].

### 2.2. IRE1 Signaling

Inositol-requiring enzyme 1α (IRE1 α) and inositol-requiring enzyme1β (IRE1 β) are two paralogues of IRE1 showing significant sequence homology in humans. IRE1α is ubiquitously expressed and IRE1β is mostly present in the gastrointestinal tract and the pulmonary mucosal epithelium. IRE1 is an ER membrane-embedded biosensor that is bound to the BiP chaperone protein, which keeps them in an inactive state. Upon accumulation of misfolded or unfolded proteins, BiP becomes detached from IRE1 and binds unfolded proteins, making the sensor active. Activated IRE1 further oligomerizes, trans-autophosphorylates, and causes the activation of its cytosolic kinase domain. Phosphorylation of IRE1 occurs at Ser724, Ser729, and Ser726 positions [22]. This phosphorylation is important for the recruitment of tumor necrosis factor receptor-associated factor 2 (TRAF2), which activates apoptosis signal-regulating kinase 1 (ASK1), thus activating c-Jun N-terminal kinase (JNK) pathway signaling [13]. IRE1α possesses an endonuclease activity for mRNA and splices the “*X-box binding protein* (*XBP1*)” transcript. In normal conditions, ribosomes cannot bind to the unspliced *XBP1* transcript because of the presence of an intron formed of a 26-nucleotide-long dual-stem loop. Once *XBP1* mRNA is spliced by IRE1, the ribosomes can bind and translate it as a functional protein called XBP1s. XBP1s translocates into the nucleus and acts as a transcription factor through its transactivation domain by inducing the expression of various UPR genes, chaperones, and ERAD pathway-related genes. Activated IRE1 also degrades all transcripts that try to enter the ER lumen by a process called RIDD (regulated IRE1-dependent decay), thus reducing the protein folding load on the ER and thereby restoring protein homeostasis [23]. In 2013, it was demonstrated that miR-1291 binds to the IRE1 transcript 5′-untranslated region (UTR) and mediates *IRE1* mRNA degradation. As a consequence of IRE1 silencing by miR-1291, glypican-3 (GPC3) protein was up-regulated. It was shown that the ER sensor IRE1 cleaves *GPC3* mRNA through a canonical cutting site located in its 3′-UTR and thus negatively regulates *GPC3* expression by destabilizing its mRNA [24]. In summary, the XBP1s protein plays key physiological and pathophysiological roles through the regulation of various metabolic pathways, genes, and cellular activities.

### 2.3. ATF6 Signaling

Like IRE1α and PERK, when the BiP protein detaches from ATF6 and binds to misfolded/unfolded proteins, ATF6 becomes activated and is translocated from the ER membrane to the Golgi through its Golgi localization sequence [25]. ATF6 is then recognized by two resident Golgi proteases, namely site-1 protease (S1P) and site-2 protease (S2P), which cleave an ATF6 domain called the “intra-membrane domain”. The cleaved ATF6 then acts as a transcription factor, migrates to the nucleus, and promotes the expression of *XBP1*, *HSPA5*, and various genes encoding ER-localized chaperones to promote protein folding and ER homeostasis recovery [26]. Thus, activated ATF6 predominantly promotes cell survival.

## 3. ER Stress Response in Liver Diseases

Owing to the high capacity of synthesizing proteins inside the ER of hepatocytes, it signifies that UPR plays an important role in mediating pathological changes in various liver diseases. Hepatocytes also perform various metabolic functions, including the biosynthesis of cholesterol and the assembly and secretion of various lipoproteins. They also play an important role in xenobiotic metabolism [27]. Disturbance in ER homeostasis leads to activation of the UPR sensors, which occurs in various liver diseases. This phenomenon may be due to the generation of reactive oxygen species, which leads to oxidative stress and hence can alter the machinery of hepatocytes. ER dysfunctions are associated with several pathological conditions, including viral hepatitis, non-alcoholic fatty liver disease, protein conformational disease, cholestasis, hyperhomocysteinemia, hepatocellular carcinoma, and many more. Despite the research on the up-regulation or down-regulation of ER stress signaling in various liver diseases, the exact role of the ER stress response in many liver pathologies remains to be fully determined. In this review, we review the well-established relationships between ER stress, UPR signaling, and liver diseases.

### 3.1. ER Stress and Metabolic (Dysfunction) Associated Fatty Liver Disease (MAFLD)

Metabolic (dysfunction) associated fatty liver disease (MAFLD) is an extensively studied liver disease. The risk factors for MAFLD are obesity, insulin resistance, hypertension, and hypertriglyceridemia. MAFLD comprises many liver disorders such as non-alcoholic fatty liver (NAFL), simple hepatic steatosis, and non-alcoholic steatohepatitis (NASH), which is the inflammation of hepatocytes. In most cases, patients are diabetic and do not undergo cirrhosis. NASH has characteristics of inflammation, which generally progress into fibrosis and eventually cirrhosis. Most patients with NASH are obese adults. There is a direct relationship between ER stress and fatty liver, as ER plays a crucial role in fatty acid synthesis and in cholesterol metabolism. Hence, chronic ER stress may promote fatty liver disease. The role of ER stress in MAFLD has been studied in various models. ER stress can lead to the production of various oxidative stress markers, and the activation of downstream NF-κB (nuclear factor kappa-light-chain enhancer of activated B cells) signaling. ER stress also causes steatosis by disrupting lipid secretion. However, lipid secretion, a high-fat diet (HFD), and insulin resistance may cause ER stress [28]. Insulin resistance is characterized by the failure of insulin to promote glucose uptake and plays a key role in suppressing glucose metabolism in the liver. Impaired insulin signaling can disturb the glucose and lipid metabolism and thus disturb the ER homeostasis. HFD can cause an increased influx of lipid and fatty acids in the liver and adipose tissue causing lipotoxicity. Protein kinase B (AKT) is a serine-threonine kinase that acts as an important target of insulin signaling and inhibits the uptake of hepatic glucose. Tribble’s homolog 3 (TRB3) is induced by ER stress through ATF4 and CHOP. TRB3 is highly expressed in the liver of diabetic mice and inhibits the activity of AKT, hence disrupting insulin signaling. JNK-mediated inhibition of insulin receptor substrate-1 (IRS-1) leads to insulin resistance and causes fatty liver. It can thus be concluded that owing to TRB3 and JNK, resistance to insulin causes an increase in insulin and in hepatic lipogenesis. ER stress activates various intracellular pathways, which can lead to the activation of NRF2, cyclic adenosine monophosphate (cAMP)-responsive element-binding protein H (CREBH), CHOP, JNK, NF-κB, inflammation, and consequently, disease progression. Phosphorylation of eIF2α leads to an increased expression of genes that cause lipogenesis and worsen liver steatosis [15]. NF-κB acts as a double-edged sword in MAFLD. Mild increases in the level of NF-κB can protect liver cells from death while excessive up-regulation can cause NASH due to excessive inflammations [29]. Insulin-induced gene 1 protein (INSIG1) is a transmembrane protein found inside the ER that plays an important role in the metabolism of cholesterol, lipogenesis, and glucose homeostasis. It controls the activation of sterol regulatory element-binding proteins (SREBPs) by binding SREBP cleavage-activating protein (SCAP) and HMG-CoA (3-hydroxy-3-methylglutaryl-coenzyme A) reductase (HMGR) and by transporting them to the Golgi, where they undergo regulated intra-membrane proteolysis (RIP) and trigger lipid biosynthesis [30,31]. ER stress also causes lipogenesis by activating caspase 2, which functions as a potential NASH biomarker [32]. In the presence of cholesterol, SCAP undergoes conformational changes which help it to bind through INSIG1 and thereby to prevent translocation to the Golgi. When hepatocytes become stressed, protein synthesis is shut off through a PERK-mediated mechanism and the intracellular INSIG1 protein level is decreased, leading to a reduction in the translocation of SREBPs to the Golgi and lipogenesis [33]. Translational inhibition may also occur through the PERK-mediated process, which also causes ERAD.

Some studies have reported the increased accumulation of fatty acids or the deposition of liver triglycerides in an insulin resistance state, thereby causing ER stress [34]. Conversely, it might be ER stress which causes the accumulation of fatty acids or triglycerides inside hepatic cells [34]. Hepatic steatosis and increased secretion of apolipoprotein B (apoB), especially very low-density lipoprotein (VLDL), cause both insulin resistance and ER stress [35]. This ER stress was accompanied by an increase in BiP/GRP78 and increased phosphorylation of eIF2α and PERK in the livers of obese mice, which means that obesity is a signal of ER stress and might inhibit the translation and transport of apoB [36]. Some studies also suggest that tunicamycin, which is a potent ER stress inducer that further activates UPR, reduces the secretion of apoB [37]. Glucosamine-induced ER stress also decreases apoB secretion from the liver [38]. Hence, increased lipogenesis and a decrease in apoB cause ER stress as a consequence of fatty acid accumulation.

IRE1 plays an important role in hepatic steatosis. Several enzymes involved in the synthesis of fatty acids and triglycerides, such as stearoyl-CoA desaturase and acetyl CoA carboxylase 2, are regulated by XBP1s protein. As a consequence, the deletion of the *XBP1* gene from the liver causes hypo-cholesterolaemia and hypotriglyceridaemia in mice and they no longer suffer from liver steatosis, even when placed on a HFD [39].

### 3.2. ER Stress and Primary Biliary Cirrhosis

Cholestatic liver disease or primary biliary cirrhosis (PBC) is a chronic liver disease that occurs due to biliary obstruction, which further causes disturbance in bile formation and biliary flow. An increase in some ER stress markers like BiP/GRP78 has been observed in patients with PBC [40]. A body of evidence suggests that the accumulation of toxic bile acids in the liver induces ER stress. Bile acids and deoxycholic acids cause the accumulation of unfolded or misfolded proteins [41]. An up-regulation of BiP, phosphorylated IRE1, eIF2α, and CHOP has been observed in hepatocytes treated with various bile acids. Moreover, there is a link between ER stress and regulated autophagy in PBC. Autophagy enhances the process of bile duct lesions and also mediates the process of biliary epithelial senescence [42]. In a previous study, it was shown that hepatic cyst formations occur due to disturbed primary cilia and originate from underdeveloped bile ducts [43]. By whole genome sequencing, it was found that cyst formation in a mutant Zebrafish phenotype occurs due to missense mutations in the *FURIN* gene, which encodes for a proprotein convertase. This mutation causes a change in Furin protein localization and ER stress [44].

Forkheadbox (FOX) is a family of transcription factors that plays an important role in the development of the liver, brain, pancreas, and lungs. FOX family members include FOXA1, FOXA2, and FOXA3. FOXA2 participates in liver development [45]. In mice, *FOXA2* silencing caused defective liver development, and *FOXA2* knockout mice were highly sensitive to a diet containing cholic acid, as illustrated by an accumulation of bile salts in the liver, which also causes ER stress [46].

### 3.3. ER Stress and Alcoholic Liver Disease (ALD)

ALD induces hepatic lesions, steatosis, and cirrhosis. Steatosis or inflammation may cause mild liver damage, while severe alcoholic hepatitis can cause cirrhosis, liver failure, or hepatocellular carcinoma. Various pathophysiological reasons may account for this disease, including chronic cell injury, oxidative stress, and inflammation [47]. Alcohol consumption triggers the accumulation of ROS and consequently ER stress. Oxidative stress is caused mainly by alcohol-induced cytochrome p450 2E1 (CYP2E1), which induces an ER stress response. Acetaldehyde induces ER stress by activating SREBP-1 in hepatic cells in all parts of the liver. SREBP-1 acts as a master regulator in lipid biosynthesis and metabolism and can cause excess accumulation of lipids within the ER inducing ER stress. It can also disturb cholesterol homeostasis, thereby exacerbating ER stress via regulating PERK [48]. ER stress was observed in various models of alcohol-induced hyper-homocysteinemia and it led to an up-regulation of lipid synthesis and, in some conditions, to cell apoptosis [49]. The accumulation of free fatty acids and triglycerides rich in lipid droplets in hepatic cells is a major cause of ALD. The deposition of free fatty acids in liver cells induces ER stress by activating ATF4, which in turn decreases lysosome-associated membrane protein 2 (LAMP2) expression and leads to a disturbance in autophagy flux [50]. Free fatty acids activate the mechanistic target of the rapamycin complex 1 (mTORC1)-ATF4-CHOP pathway in ALD [51].

The role of ER stress in ALD has been investigated in various animal models by feeding them with alcohol and by observing whether they develop chronic liver inflammation and, later, severe steatosis. As a consequence, SREBP-1c is up-regulated and was found to protect mice against the excessive deposition of triglycerides [52]. In that study, lipogenic enzymes like fatty acid synthase, acetyl CoA carboxylase, and stearoyl-CoA desaturase were also elevated. If the level of phosphorylated eIF2α is low, UPR is mildly activated, while activation of PERK, IRE1, and ATF6 is a sign of severe UPR activation. ER stress causes inflammation by activating the NF-kB and JNK pathways. ATF4 expression is also up-regulated in alcohol-induced liver injury by activating the AMP-activated protein kinase (AMPK) signal [53]. In chronic conditions, the levels of BiP, calcium, and CHOP are high and lead to cell apoptosis, and ER homeostasis is profoundly disturbed as a result of increased amounts of ER oxidoreductase 1, BCL2 associated X-protein (BAX), and calnexin. Increased SREBPs disturb the lipid metabolism by promoting the production of triglycerides and cholesterol [54]. The nuclear factor of activated T cell (NFAT) proteins contains a family of calcium responsive transcription factors (NFATc1-NFATc4), particularly NFATc1,that sense ER stress and cause the activation of PERK-CHOP, thus signaling worsening liver damage [55].

### 3.4. ER Stress and Viral Hepatitis

Viral hepatitis, which mostly comprises hepatitis B virus (HBV) and hepatitis C virus (HCV), causes chronic inflammation and if left untreated, may lead to fibrosis, cirrhosis, and liver cancer. When viral infections occur in the host cells, it produces and utilizes a large number of viral proteins that overloads and imbalances ER homeostasis, leading to ER stress if not controlled [56,57]. UPR signaling activation has been observed in vitro and in vivo following viral hepatitis infection [58]. Expression of the Interferon-***γ***3 and Interferon-***γ***4 (IFN-***γ***3/IFN-***γ***4) has been associated with a high risk of chronic HCV. It was also reported that patients with the *IFNL4* genotype have a reduced risk of cirrhosis because of a high ER-stress induction due to the accumulation of the IFN-λ4 protein, which has anti-proliferative and pro-apoptotic effects [59].

Apolipoprotein H (apoH) is an HBV protein that has the potential to bind hepatitis B surface antigens (HBsAg) and to inhibit its secretion. ApoH is highly regulated by HBV and promotes ER stress as a result of intracellular retention of the hepatitis B virus and chronic infection [60]. Non-secreted HBsAg accumulates in cells and causes ER stress, and consequently UPR signaling activation and hepatocyte transformation [56]. In line with previous data, the expression of both isoforms of AFT6 proteins was significantly higher in HCV-mediated fibrosis compared to normal livers [57]. Moreover, activation of PERK consecutive to phosphorylation on Thr980 results in phosphorylation of eIF2α on Ser21 in diseased liver compared to normal liver. Similarly, eIF2α was more phosphorylated on Ser21 in fibrosis conditions than in normal conditions. In HCV-related fibrosis there was a significant increase in the mRNA levels of Ki67, JUN which leads to cell inflammation and proliferation. There was up-regulation of cAMP responsive element binding protein 3 like 3 (CREB3L3) and immediate early response 3 (IER3S) (which encodes pro-apoptotic immediate early response 3 short variants) which further support apoptosis induction. A preliminary study showed that HBV also activates the ATF4 pathway to induce cyclooxygenase-2 (COX2) and cause inflammation [58], while another study showed that the interaction of HBX protein with GRP78 results in the suppression of eIF2α phosphorylation and the reduction of ATF4, CHOP, and poly-ADP ribose polymerase (PARP) protein, thus preventing cells from engaging in an apoptotic process [61].

### 3.5. ER Stress and Hepatic Ischaemia-Reperfusion Injury

Hepatic ischaemia-reperfusion injury (IRI) is a pathophysiological condition that affects the liver and other organs, and it often causes irreversible damage to the tissues. Several factors may trigger it, including oxidative stress, overload of intracellular calcium, the secretion of cytokines and chemokines by liver Kupffer cells, and anaerobic metabolism. Hepatic IRI occurs mainly in the donor’s liver during liver transplantation. ER stress and UPR signaling play an important role in this injury. A study reported the role of vitamin D receptors (VDR) in inflammation and IRI injury and its connection with ER stress and autophagy through the up-regulation of CHOP [62]. Up-regulated expression of BiP, CHOP, spliced XBP1 phosphorylated PERK and eIF2α occurred in the diseased liver. Conversely, cytoprotective proteins such as Bax inhibitor-1 (Bl-1), which suppresses cell death, were also induced. Loss of Bl-1 causes the up-regulation of IRE1α and ATF6 and increases liver damage. A study using knockout mice models suggested that BI-1 plays a protective role against ER stress and IRI in the liver. BI-1 knockout mice exhibited severe histological injury, higher levels of the liver enzyme in the bloodstream showing more liver cell death, increased caspase activity, and higher expression of proteins involved in ER stress response compared to wild-type mice with intact BI-1. It can be concluded that BI-1 plays a critical role in protecting the liver against ER stress during IRI [63]. Finally, it was shown that antibiotics improve IRI-stressed tissues in mice by limiting inflammation through the decrease in CHOP and the increase in autophagy [64]. A novel role of macrophage thioredoxin-interacting protein (TXNIP) is induced in liver inflammation and has been found to be involved in mediating cylindromatosis (CYLD)–NRF2–OASL1 axis. When there is an absence of TXNIP in macrophages, it enhances the activity of CYLD which is involved in controlling inflammations and liver injury due to stress. It further regulates NRF2 and OASL1. This axis is a critical regulator of stimulators of interferon genes/tank binding kinase 1 (STING/TBK1) mediated immune response and also mediated apoptotic pathways in IR stress liver injury [65]. Hyperglycemia triggers ER stress, thereby activates ATF6-CHOP pathways and inhibits β-catenin, causing deteriorating liver in hepatic IRI [66].

### 3.6. ER Stress and Alpha-1 Antitrypsin Deficiency

Alpha-1 antitrypsin (AAT) is produced mainly by hepatocytes in the liver, but it is also produced in small amounts in monocytes, alveolar cells, macrophages, and intestinal epithelium [67]. In the lungs, AAT acts as an antiprotease protective screen in which it neutralizes the activity of serine protease, neutrophil elastase, proteinase 3, and cathepsin G [68,69]. AAT deficiency is a genetic disorder that results from a homozygous “Z” mutation in the *SERPINA1* gene which encodes AAT. The Z allele of *SERPINA1* generates a mutated AAT protein called Z-AAT. It mainly occurs in the liver following the accumulation of unfolded or misfolded Z-AAT proteins in the ER of hepatocytes. However, it also causes obstructive pulmonary diseases owing to the absence of protease inhibitor activity in the lungs [67]. *SERPINA1* mutations occur at position 342 where a glutamic acid is replaced by lysine [70]. CHOP and c-JUN causes up-regulation of *SERPINA1* and hence worsens the disease [71]. The deposition of Z-AAT protein in the liver causes liver damage and may further evolve into cirrhosis and hepatocellular carcinoma. Some studies showed that there is no significant activation of UPR signaling in AAT deficiency, yet there is specific activation of caspase 12, caspase 4, and the NF-κB pathway [72]. Some researchers hypothesized that Z-AAT proteins accumulate only when cells are exposed to an ER stress inducer. This promotes the interaction of Z-AAT protein with IRE1 and further causes ATF6 signaling activation [72,73]. Moreover, the increased expression of ATF4, ER chaperones, and ERAD has also been documented and the activation of ATF6 signaling attenuates Z-AAT protein accumulation and promotes the ERAD pathway [74]. It has also been found that protein disulphide isomerase, particularly PDIA4, is up-regulated in cases of AAT deficiency (AATD) [75].

### 3.7. ER Stress and Hepatocellular Carcinoma

Hepatocellular carcinoma (HCC) is a frequent primary cancer of the liver that usually arises from NASH, viral hepatitis, alcohol abuse, and aflatoxin ingestion [76]. Hypernutrition in liver parenchymal cells eventually results in NASH, which in turn leads to HCC by an inflammatory mechanism that depends on TNFβ and IκB signaling pathways. ER stress is usually observed in many cancers. Some studies suggest that mild ER stress does not trigger hepatocarcinoma in mice fed on a low-fat diet. However, mice placed under extensive oxidative stress with hyper nutrition develop NASH and eventually HCC [77].

A few studies also suggest a relationship between HFD, ER stress, and the onset of HCC [78,79,80,81]. Mild ER stress does not trigger hepato-carcinogenesis when mice are kept on a low-fat diet. However, when treated with hyper nutrition, they spontaneously develop NASH which progresses to HCC [82]. This could be due to lipid droplet accumulation, SERBP1 activation, or increased ROS production, which may trigger oncogenic mutations and/or genomic instability. In parallel, the release of inflammatory mediators and TNFβ is enhanced, and this activates tumor-associated macrophages which promote the growth of HCC progenitors [78]. UPR promotes hypoxia conditions which encourages tumor cells to survive in hypoxia conditions. During hypoxia conditions, splicing of XBP1 occurs which promotes tumor formation [83]. ATF6, XBP1s, and BiP are highly expressed in patients with HCC. Phosphorylated PERK, eIF2α, and TRAF2 are also increased in the later stages. CHOP also plays a role in ER stress-induced HCC apoptosis [18]. Therefore, targeting ER stress is a promising therapeutic option in the treatment of HCC. For instance, inhibiting IRE1 endonuclease activity decreases tumor development in mice with HCC [78]. However, the molecular crosstalk between ER stress and HCC development remains unclear.

### 3.8. ER Stress and Drug-Induced Liver Injury

Several drugs may eventually cause injury to the liver. Acetaminophen induces severe liver injury when overdosed by triggering glutathione depletion from the ER, the phosphorylation of eIF2α and JNK, and inducing the transcription factor GADD34 and CHOP [84]. Other drugs like cyclosporine A and arylating quinone also induce ER stress and liver injury [85]. Efavirenz, an antiviral drug, causes up-regulation of CHOP and GRP78 mRNA and protein expression, phosphorylation of eIF2α, and up-regulation of XBP1s [86]. Rifampicin, an antituberculosis drug, causes liver injury and also activates ER stress [87]. Cyclosporine A causes cholestasis, while arylating quinine induces phosphorylation of eIF2α and PERK and activates ATF4 and CHOP [88,89]. Quinones have the potential to generate ROS and contribute to homeostasis as well as cytotoxicity. Studies have demonstrated a connection between cyclosporine A-induced cholestasis and endoplasmic ER stress. Cyclosporine A treatment in mice increases mmu-miR-182-5p levels, which in turn down-regulate the expression of genes involved in the synthesis of bile acid and its transport, leading to cholestasis. These findings pave the way for potential therapeutic targets for the treatment and prevention of cyclosporine A-induced cholestasis [2,90].

### 3.9. UPR Mediated Apoptosis

Prolonged ER stress can trigger pro-apoptotic signaling via IRE1, PERK, and ATF6 by not directly causing cell death but rather by activating downstream molecules which drag cells towards cell death. CHOP, which is up-regulated by all three arms of UPR, induces apoptosis by increasing the expression of target genes like *BCL2, GADD34, TRB3*, and endoplasmic reticulum oxidoreduction 1 (*ERO1*) [91]. It also induces the expression of Bim and PUMA (p53 up-regulated modulator of apoptosis) proteins which are pro-apoptotic proteins that favor the activation of apoptotic pathways [92]. It has been found that the hepatic levels of PUMA are increased in patients with NASH and this plays an important role in apoptosis [93]. GADD34 can dephosphorylate the phosphorylated eIF2α by binding the α-subunit of protein phosphatase 1 and thereby continue the translation of proteins causing increased ER stress and triggering cells towards apoptosis. Apoptosis can be induced by IRE1α-TRAF2-JNK pathways [94]. Apoptosis occurs in hepatic cells by the CHOP-ERO1α pathway, mediated by caspase 12 [95].

## 4. Therapeutic Approaches

Dysregulated protein degradation can be used as a therapeutic target in the treatment of various liver diseases (Table 1).

Increased deposition of Z-AAT proteins induces a serious liver pathology called AAT deficiency (AATD). Targeting this ER-dependent mutated protein by gene edition is a promising therapeutic strategy [112]. Several drugs like rapamycin, 24-norursodeoxycholic acid (Nor UDCA), and carbamazepine (CBZ) have been used in clinical trials and were found to significantly decrease the accumulation of mutant Z-AAT proteins by inducing autophagy. Inhibiting protein disulphide isomerase (PDI), particularly PDIA4, can be a potent therapeutic approach in AATD patients [75]. Some anti-diabetic drugs like metformin are potential therapeutic targets for MAFLD [113,114]. Mice treated with empagliflozin along with HFD showed a significant decrease in MAFLD. The progression of inflammation was slowed and HFD-induced ER stress was reduced by a decrease in the expression of GRP78, IRE1α, XBP1s, phosphorylated eIF2α, CHOP, and ATF6. Empagliflozin also improved fasting blood glucose and lipid levels and attenuated hepatic apoptosis. It also reduced the levels of lipogenic enzymes and the expression of pro-inflammatory markers [98]. Inhibiting caspase 2 inhibition may be effective for treating stress-related fatty liver diseases [61,115]. In addition, berberine, an isoquinoline alkaloid isolated from medicinal herbs from China, is used in the treatment of MAFLD. Berberine inhibited the activation of PERK-ATF4-CHOP signaling in murine hepatocytes and macrophages. However, it had no effect on the levels of ATF6, IRE1α, and GRP78 proteins [116]. It also improved liver injury induced by glucose and lipid metabolic disorders [116]. Exogenous H_2_S have been found to reduce gene expression and can be used for therapeutic potential to improve hepatic IR damage [117].

Hepatic cysts can be treated by ER stress inhibitors like 4-phenyl butyric acid or by mTOR inhibitors such as rapamycin [44]. It has also been found that polyunsaturated fatty acids (PUFA) have anti-inflammatory properties which protect liver cells from the toxicity induced by bile acids [118].

Ursodeoxycholic acid (UDCA) plays an important role in treating cholestasis [103]. Patients who are intolerant to UDCA can be treated with obeticholic acid (OCA), which is a farnesoid X receptor agonist [119]. When PUFA was administered together with UDCA, the combination prevented bile acid-induced apoptosis and ER stress [120]. Suppressing the activity of mTORC1 with rapamycin down-regulates ER stress due to the accumulation of free fatty acids, increases the level of LAMP2 protein, and limits the deleterious effects of ALD [51].

IRE1α RNase activity can be used as a treatment for IRI [121]. A study performed in mice treated with antibiotics showed a significant improvement in IRI-stressed cells by decreasing CHOP, increasing autophagy, and inhibiting the pro-inflammatory response. Interestingly, the microbiome present in the gut can be used as a potential target to improve liver transplants [64]. VDR can be used as a potential self-defensive mechanism for hepatic IRI by enhancing autophagy and reducing the inflammatory response [122].

A study using a mouse model with HFD feeding for 12 weeks, then CBZ or rapamycin every other day during the last week of feeding, showed a significant decrease in the accumulation of lipid droplets and plasma insulin, and a decrease in hepatic and serum triglycerides [105]. Other studies reported that treating ALD patients with rapamycin decreases the toxicity of ethanol exposure by enhancing the degradation of lipid droplets. Rapamycin can also be used for treating hepatosteatosis [123].

Some molecules like sinulariolide, which initiates hepatic apoptosis in vitro, have been used to block HCC development [109]. Tripartite motif-containing (TRIM) family proteins, especially TRIM25, are up-regulated in oxidative stress which promotes ER-associated degradation by targeting Nrf2-Keap1 pathways resulting in reduced IRE1 signaling [124]. PERK inhibitors GSK2656157 and GSK2606414 not only inhibit PERK signaling but also receptor interacting protein kinase 1 (RIPK1) which can be used for targeting PERK in HCC cases [125]. C16, an inhibitor of RNA-dependent protein kinase, is used for targeting MAFLD in vitro [100]. It is also called a PKR (Protein Kinase R) inhibitor. NRF2-mediated SIRT3 induction protects liver cells from ER stress injury [32]. Fibroblast growth factor 21 (FGF21), a stress-inducible hormone, plays an important role in regulating glucose and lipid homeostasis. It is used to treat drug-induced liver injury and works by inhibiting eIF2α, ATF4, and CHOP activities [106]. Apigenin, a common food flavonoid, prevents SREBP-2 activation by modulating its nuclear translocation [126,127]. Mycotoxins inhibit *XBP1* mRNA splicing [127]. Geldanamycin is a naturally occurring compound which has anticancer properties and helps in inhibiting the growth of cancer cells. It binds to GRP94, an HSP90-like protein specialized in the folding and processing of proteins found inside the ER, and inhibits its chaperone’s activity. As a result, the proteins which depend on GRP94 for proper folding are degraded. Thus, it can be concluded that geldanamycin can be potentially used as a therapeutic drug for treating cancers [126]. Finally, MKC-3946 is a small molecule inhibitor of IRE1α that can be used as a therapeutic agent for treating cancer. It selectively targets the IRE1α-XBP1 pathway and inhibits the activity of IRE1α, causing a decrease in cell viability [111].

As shown in this section, many drugs and treatment options have been tested and validated in laboratories to limit the development of liver disorders and to treat patients. However, a very limited number of drugs have reached the clinical stages and demonstrated any therapeutic benefit for patients with metabolic or malignant liver pathologies.

## 5. Discussion

**Figure 2:**
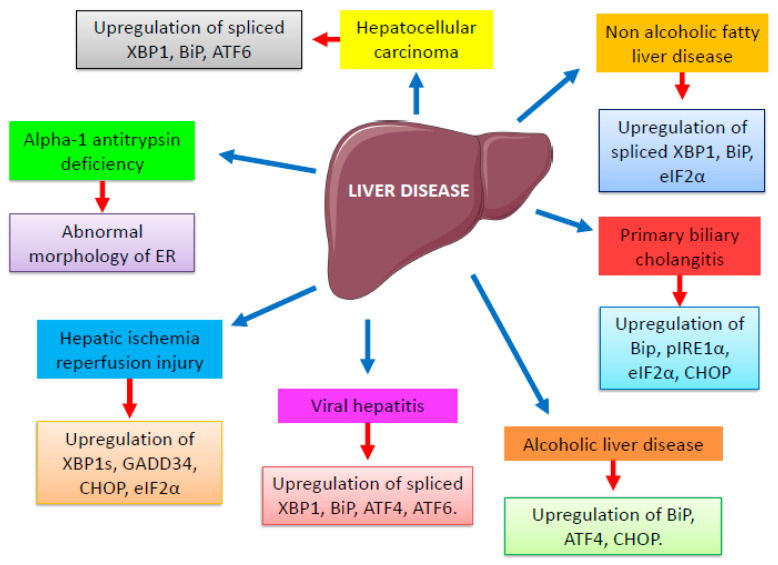
Role of various UPR markers in liver diseases. Figure 1 and Figure 2 were partly generated using Servier Medical Art, provided by Servier (Suresnes, France), licensed under a Creative Commons Attribution 3.0 unported license.

As a result of various liver injuries, the ER stress-induced response has the potential to activate several signaling pathways and produce multiple pathophysiological changes. UPR signaling acts as a double-edged sword, i.e., it contributes to the improvement of the conditions of the disease or counteracts them depending on the impact and intensity of ER stress and the mobilized sensors or mediators. UPR signaling not only maintains ER homeostasis, but it is also closely connected to the control of glucose and lipid metabolisms. Activation of UPR signaling leads to the dissociation of BiP from IRE1α, PERK, and ATF6. Activated PERK induces the phosphorylation of eIF2α, which inhibits the overall protein translation. In parallel, it causes an elevation of ATF4 which is translocated into the nucleus and induces the expression of various target genes, such as ERAD for protein degradation and CHOP for apoptosis. Again, activation of IRE1α induces the splicing of *XBP1* mRNA and translation of the XBP1s protein, which in turn promotes gene expression. Activated ATF6 is transported to the Golgi and induces proteolytic cleavage, in turn activating a specific set of genes including *ERN1*, *ATF6*, *XBP1*, *DDIT3*, and *HSPA5*. The involvement of various UPR pathways and their targets in the different liver disorders is represented in Figure 2. The pharmacological effects of ER stress and UPR activation in liver disease need to be tested in greater depth to discover novel effective targets to treat various liver diseases. ER stress mediators can serve as biomarkers for liver diseases. Increased levels of specific UPR genes in patient samples can be used as identification for the severity of ER stress and can also be linked with disease progression. For example, high expression of spliced XBP1 was found to be associated with distant metastasis and poor prognosis in HCC [128]. These ER stress markers can be used for categorizing patients with different risks. These ER stress mediators along with some clinical parameters can enhance prognostic accuracy.

UPR is induced in hepatic cells in response to high-fat diets, viral infection, drugs, and ROS. When there is mild ER stress, these signaling pathways can be protective by trying to maintain the normal physiological functions of the liver, but they can drive cells towards apoptosis if stress is prolonged. In this review, we have tried touching on the various aspects of UPR in liver diseases. The exact and comprehensive role and mechanism of ER stress in contributing to liver disease and the specific involvement of individual UPR pathways are partially known. UPR-associated biomarkers can be useful for the prognosis of liver disorders. Currently, UPR activation during organ transplantations is gaining attention, and targeting these UPR pathways can reduce organ damage during organ transplantations. Further research on the role of UPR in liver diseases may be helpful for finding new therapeutics in liver disorders.

## Figures and Tables

**Table 1:** Drugs and their targets used in various liver disorders.

Disease	Drugs/Treatment Options	Working Action	Clinical Trials	References
Metabolic (dysfunction) associated fatty liver disease (MAFLD)	Anti-diabetic drugs (metformin)	Decrease GRP78, IRE1α, XBP1s, eIF2α, CHOP, ATF6.	NCT00063232 with published data	[96,97]
	SGLT-2 inhibitors	Inhibit de novo lipogenesis, oxidative response	Recruiting or completed, but no available data	
	Empagliflozin	Increases autophagic flux	Recruiting or completed, but no available data	[98]
	Isoquinoline (berberine)	Inhibits PERK-ATF4-CHOP axis	Recruiting or completed, but no available data	[99]
	C16	PKR	/	[100]
	Salubrinal	Inhibitor of eIF2α dephosphorylation	/	[101]
	Dantrolene	Reduces calcium leakage from ER	/	[102]
Cholestasis	Ursodeoxycholic acid	Unfolded protein response signaling	Drug used in clinical practice since 1990s	[103]
Alcoholic liver disease	Rapamycin	autophagy	/	[51]
Hepatic ischemia-reperfusion injury	Antibiotics	Autophagy	/	[104]
	Vitamin D receptors	Autophagy	/	[47]
	Rapamycin	Autophagy	/	[105]
Drug induced liver injury	FGF21	eIF2α, ATF4, CHOP	/	[106]
	Apigenin	ATF6	/	[107]
Alpha_1_-antitrypsin deficiency	Carbamazepine	Autophagy	/	[108]
Hepatocellular carcinoma	4-phenyl butyric acid		/	[109]
	Berberine		/	[99]
	Geldanamycin	Target GRP94	/	[110]
	MKC-3946	IRE1α inhibitors	/	[111]

## Abbreviations

AATD	Alpha-1 antitrypsin (AAT) disease
ALD	Alcoholic liver disease
apoH	Apolipoprotein H
ARE	Antioxidant response element
ATF4	Activating transcription factor 4
ATF6	Activating transcription factor 6
BCL2	B-cell lymphoma 2
CHOP	C/EBP-homologous protein
eIF2α	Eukaryotic translation initiation factor 2α
ER	Endoplasmic reticulum
ERAD	ER-associated degradation
FGF21	Fibroblast growth factor 21
GADD34	Growth arrest and DNA damage-inducible protein 34
HCC	Hepatocellular carcinoma
HFD	High-fat diet
IRE1	Inositol-requiring enzyme 1
MAFLD	Metabolic (dysfunction) associated fatty liver disease
NASH	Non-alcoholic steatohepatitis
PDI	Protein disulfide-isomerase
PERK	Protein kinase RNA-activated (PKR)-like ER kinase
ROS	Reactive oxygen species
UPR	Unfolded protein response
XBP1	X-box-binding protein 1
XBP1s	Spliced XBP protein 1
